# Primary sclerosing cholangitis with increased immunoglobulin G4 levels: A case report

**DOI:** 10.1097/MD.0000000000018411

**Published:** 2019-12-16

**Authors:** Qing-Ling Chen, Rui Zhong, Xiao-Xue Zhang, Li-Na Feng, Xiao-Yu Wen, Qing-Long Jin

**Affiliations:** a Department of Hepatology; b Department of Neurology, The First Hospital of Jilin University, Changchun, Jilin, China.

**Keywords:** case reports, cholestasis, immunoglobulin G4, sclerosing cholangitis

## Abstract

**Rationale:**

Primary sclerosing cholangitis (PSC) is recognized as an autoimmune-mediated liver disease characterized by progressive biliary inflammation and fibrosis. Some PSC cases with elevated immunoglobulin G4 (IgG4) levels are likely to be misdiagnosed with immunoglobulin G4-related sclerosing cholangitis (IgG4-SC). Thus, distinguishing these 2 diseases is particularly important.

**Patient concerns:**

A 34-year-old male presented with right hypochondrium abdominal intermittent pain and jaundice lasting for 1 month. Here, we present a case of PSC with increased IgG4 levels with improvement of quality of life upon liver transplantation (LT).

**Diagnosis:**

The diagnosis of PSC was confirmed based on clinical symptoms, laboratory test results, imaging findings, pathologic results and a lack of response to steroid therapy.

**Interventions:**

LT surgery was performed successfully when his vital parameters were stabilized. Immunosuppressive agents were routinely used after LT.

**Outcomes:**

Three years after LT, liver function values show that alanine aminotransferase (ALT) and aspartate aminotransferase (AST) were in the normal range. An abdominal ultrasonography showed no obvious abnormalities.

**Lessons:**

There are similar biochemical characteristics and cholangiographic findings between PSC and IgG4-SC. Therefore, distinguishing these 2 diseases is particularly important. LT remains the only option for end-stage PSC. Early diagnosis and effective treatment can achieve a good prognosis.

## 1 Introduction

Immunoglobulin G4-related sclerosing cholangitis (IgG4-SC) is characterized by abundant immunoglobulin G4 (IgG4)-positive plasma cells with effective steroid therapy.^[1]^ In one study, FD Mendes et al^[2]^ discovered that 9% of primary sclerosing cholangitis (PSC) patients had elevated serum IgG4 levels. PSC, recognized as a sclerosing cholangitis without steroid efficacy, has been usually confused with IgG4-SC clinically due to similar biochemical characteristics and cholangiographic findings.^[3,4]^ Therefore, differential diagnosis between PSC and IgG4-SC is particularly significant. Here, we present a case of PSC with increased IgG4 levels with improvement of quality of life upon liver transplantation (LT). Informed consent was obtained from the patient for this publication. The ethics committee of our hospital approved this case report.

## 2 Case report

**Figure 1:**
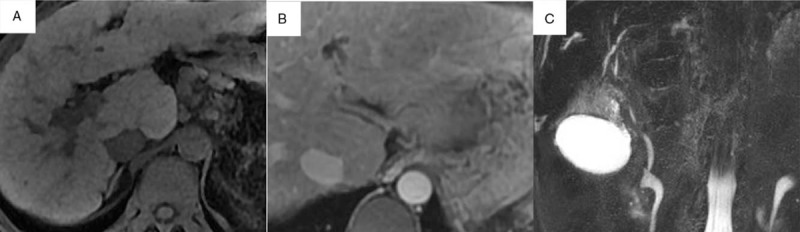
Imaging findings. A: MRI of the liver demonstrated liver cirrhosis. B: MRI of the liver showed mild dilatation in some intrahepatic bile ducts. C: Magnetic resonance cholangiopancreatography (MRCP) revealed low signal intensity in cystic ducts and obstructed bile ducts in the hepatic hilar region. MRI = magnetic resonance imaging.

**Figure 2:**
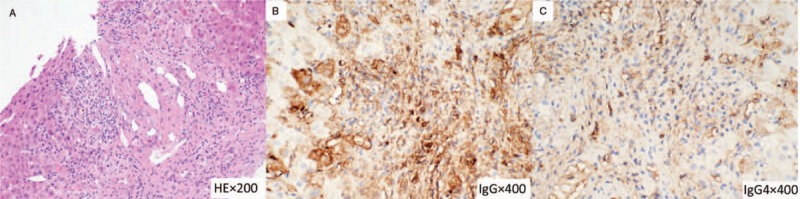
Histopathological findings of the patient before liver transplantation (LT). A: Histopathological examination by liver biopsy showed disappearance of bile ducts concomitant with arteries, proliferation of peripheral small bile ducts, fibrosis and fibrotic change surrounding the bile ducts, infiltration of abundant lymphocytes and mild levels of plasma cells, and cholestasis in peripheral hepatocytes with local biliary thrombosis in the enlarged portal area (HE staining, original magnification×200). B: IgG immunostaining of liver biopsy showed that only scattered IgG-positive plasma cells can be observed (×400). Note that the background staining was heavier. C: IgG4 immunostaining of liver biopsy demonstrated infiltration of few IgG4-positive plasma cells (1–3 cells/ hpf) (×400). Note that the background staining was heavier. HE = hematoxylin and eosin.

A 34-year-old male presented with right hypochondrium abdominal intermittent pain and jaundice lasting for 1 month. The patient had no remarkable past history including special drug or consumption of alcohol. The levels of serum IgG4 were increased in his younger sister, his daughter and the patient himself. Physical examination revealed dim and blackish complexion, jaundice, abdominal bulge, and positive shifting dullness. Note that the rebound tenderness sign and Murphy sign were negative. Laboratory results are shown in Table 1. In addition, there were no abnormalities in hepatitis B surface antigen (HBsAg), hepatitis C virus antibody (anti-HCV), anti-mitochondrial antibody (AMA), anti-nuclear antibody (ANA), smooth muscle antibody (SMA), antineutrophil cytoplasmic antibodies (ANCA), blood, and urine amylase or tumor markers. Magnetic resonance imaging (MRI) of the liver demonstrated liver cirrhosis and mild dilatation in some intrahepatic bile ducts (Fig. 1A, B). Magnetic resonance cholangiopancreatography (MRCP) revealed obstructed bile ducts in the hepatic hilar region (Fig. 1C). A liver biopsy was performed that indicated fibrosis and fibrotic change surrounding the bile ducts, infiltration of abundant lymphocytes and mild levels of plasma cells, and cholestasis in peripheral hepatocytes with local biliary thrombosis in the enlarged portal area. (Fig. 2A). IgG immunostaining of liver biopsy showed that only scattered IgG-positive plasma cells can be observed (Fig. 2B). IgG4 immunostaining of liver biopsy demonstrated infiltration of few IgG4-positive plasma cells (1–3 cells/ hpf) (Fig. 2C).

**Table 1:**
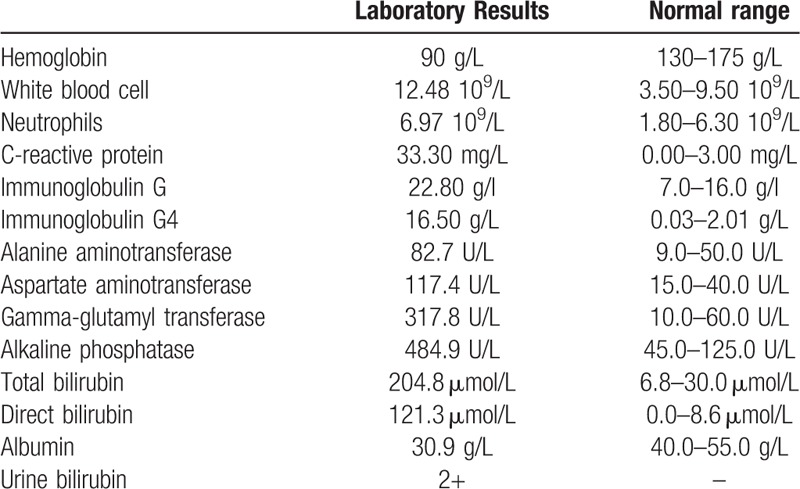
Laboratory workup at admission.

**Figure 3:**
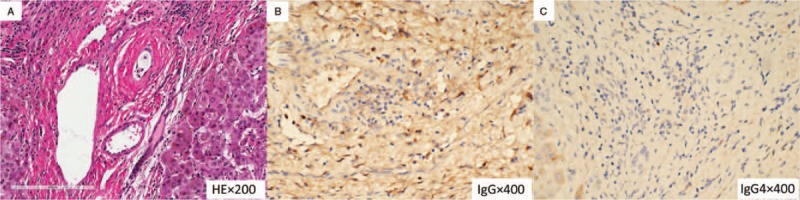
Histopathological findings of the patient during liver transplantation. A: Histopathological findings of the liver during operation showed fibrotic change, atrophied biliary epithelial cells and onion-skin fibrosis in the enlarged portal area (HE staining, original magnification×200). B: IgG immunostaining of liver showed that only scattered IgG-positive plasma cells can be observed (×400). Note that the background staining was heavier. C: IgG4 immunostaining of liver demonstrated infiltration of few IgG4-positive plasma cells (1–3 cells/ hpf) (×400). Note that the background staining was heavier. HE = hematoxylin and eosin.

At first, considering these clinical data, we suspected IgG4-SC and began oral steroid therapy at 40 mg/day. The serum IgG4 levels became normal after steroid therapy, but the jaundice did not improve. The patient's general condition gradually deteriorated, and he eventually developed severe complications after steroid therapy. Therefore, we conducted endoscopic retrograde cholangiopancreatography (ERCP), in which irregular narrowing of the intrahepatic bile ducts, that is, a pruned-tree appearance, was observed. Then, endoscopic nasobiliary drainage (ENBD) was performed. Severe esophageal and gastric varices were shown by gastroscopy. A colonoscopy confirmed chronic colitis. Thus, the diagnosis of PSC was strongly suspected based on a lack of response to steroid therapy and ERCP. Then, the patient successfully underwent LT when his vital parameters were stabilized. Histopathological findings of the liver during operation showed fibrotic change, atrophied biliary epithelial cells and onion-skin fibrosis in the enlarged portal area (Fig. 3A), IgG immunostaining of liver showed that only scattered IgG-positive plasma cells can be observed (Fig. 3B), and IgG4 immunostaining of liver demonstrated infiltration of few IgG4-positive plasma cells (1–3 cells/ hpf) (Fig. 3C), which were compatible with PSC. The diagnosis of PSC was confirmed eventually. After LT, his quality of life improved markedly.

One year after LT, the serum IgG4 levels of his younger sister, his daughter and the patient himself were above the upper limit of normal (ULN) range, as follows: 10.20 g/L, 8.86 g/L, and 11.90 g/L, respectively. HLA-DRB1∗07:01, 07:01 was found in both his younger sister and the patient himself, and HLA-DRB1∗07:01, 09:01 was found in his daughter. Three years after LT, liver function values show that alanine aminotransferase (ALT) and aspartate aminotransferase (AST) were in the normal range. An abdominal ultrasonography showed no obvious abnormalities.

## 3 Discussion

PSC is a chronic and progressive cholestatic liver disease that predominantly occurs in 30- to 40-year-old men.^[5]^ The potential etiologies of PSC have not yet been identified, which may be due to interactions among genetic and environmental factors.^[6]^ Owing to the application of genome-wide association studies, a large number of disease susceptibility genes for PSC have been confirmed.^[6–8]^ The risk of PSC in first-degree relatives of patients is increased 9 to 39 times.^[9]^ Evidence from a genome-wide association study^[10]^ indicated a significant association of HLA-DRB1∗07:01 with PSC. In addition, recent progress has found that some PSC patients with increased serum IgG4 levels also carry HLA-DRB1∗07:01.^[11]^ Based on this patient and his first-degree relatives, we hypothesize that HLA-DRB1∗07:01 may be associated with the elevated levels of IgG4 in PSC, a hypothesis that needs to be further confirmed. Approximately half of the patients with PSC have no obvious clinical symptoms in the early stage.^[12]^ Jaundice, fatigue and vague upper abdominal discomfort are most common among the symptomatic patients, who may also have gastrointestinal bleeding.^[13]^ Liver function tests suggest evidence of cholestasis, especially the elevation of alkaline phosphatase and gamma-glutamyl transferase.^[13]^ Elevated bilirubin levels indicate that the disease has developed to the middle and late stages or that dominant biliary strictures can be seen in these patients.^[5]^ The serum IgG4 levels of a small number of PSC patients may be elevated. Compared with patients with PSC with normal IgG4 levels, patients with elevated IgG4 levels had a lower frequency of inflammatory bowel disease (IBD), higher severity of this disease and shorter time to liver transplantation,^[2]^ which were compatible with this case. Typical imaging findings are extrahepatic and/or intrahepatic biliary strictures.^[4]^ MRCP has been recommended for the diagnosis of PSC, but ERCP is still essential in some dominant biliary strictures for its diagnostic and therapeutic utility.^[14]^ The only current medical therapy for PSC patients is ursodeoxycholic acid, which can improve liver enzyme levels; however, it cannot slow the progression of this disease, and LT remains the only option for end-stage PSC.^[15]^ In this case, based on clinical symptoms, laboratory test results, imaging findings, pathologic results and a lack of response to steroid therapy, the diagnosis of PSC was definitive. After LT, the patient's quality of life improved markedly.

Koyabu et al^[16]^ ever reported 3 PSC individuals with elevated serum IgG4 levels. However, our case has the following advantages. First, the efficiency and safety of LT was also apparently confirmed in PSC patients, which provided us with a warning regarding the importance of differential diagnosis with IgG4-SC and avoiding blindly administering steroid therapy. Second, we tested the HLA-DRB1∗ alleles and serum IgG4 levels for the patient's first-degree relatives and the patient himself, which provides cautionary information for clinicians to consider the role of HLA-DRB1∗07:01 in PSC with elevated IgG4 levels.

## Acknowledgments

We thank the patient and his family, who gave their informed consent for publication.

## Author contributions

**Data curation:** Xiao-Xue Zhang, Li-Na Feng.

**Formal analysis:** Rui Zhong.

**Writing – original draft:** Qing-Ling Chen.

**Writing – review & editing:** Xiao-Yu Wen, Qing-Long Jin.
